# A highly efficient protocol for isolation of protoplast from China, Assam and Cambod types of tea plants [*Camellia sinensis* (L.) O. Kuntze]

**DOI:** 10.1186/s13007-023-01120-z

**Published:** 2023-12-15

**Authors:** Abhishek Kumar, Nikhil Rawat, Shweta Thakur, Rohit Joshi, Shiv Shanker Pandey

**Affiliations:** 1 Biotechnology Division, Council of Scientific and Industrial Research (CSIR)-Institute of Himalayan Bioresource Technology, Palampur, 176061 India; 2 Academy of Scientific and Innovative Research (AcSIR), Ghaziabad, 201002 India

**Keywords:** Protoplast, *Camellia sinensis*, Enzymatic hydrolysis, Tea leaves, Tea-types

## Abstract

**Background:**

Tea is the most popular beverage worldwide second only to water. Its demand is tremendously rising due to increased awareness of its medicinal importance. The quality and uses of tea depend on the tea-types which are mainly three types including China, Assam and Cambod type having distinct compositions of secondary metabolites. Huge variation in secondary metabolites in different tea-types and cultivars limited the successful application of various approaches used for its trait improvement. The efficiency of a protocol for isolation of protoplast is specific to the types and cultivars of tea plants. The existing tea protoplast-isolation protocols [which were optimized for tea-types (China and Assam type) and Chinese cultivars grown in China] were found ineffective on types/cultivars grown in India due to type/cultivar variability. Therefore, optimization of protoplast-isolation protocol is essential for tea-types/cultivars grown in India, as it is the second largest producer of tea and the largest producer of black tea. Here, efforts were made to develop an efficient protoplast-isolation protocol from all major types of tea (China, Assam and Cambod types) grown in India and also from three types of tender leaves obtained from field-grown, hydroponically-grown and tissue culture-grown tea plants.

**Results:**

Developed protoplast-isolation protocol was effective for different types of leaf tissue obtained from the tender leaves of field-grown, hydroponically-grown and tissue culture-grown tea plants. Moreover, optimized protocol effectively worked on all three types of tea including China, Assam and Cambod types cultivated in India. The digestion of leaves with 3% cellulase R-10, 0.6% macerozyme, 1% hemicellulase and 4% polyvinylpyrrolidone for 12 h at 28ºC yielded approximately 3.8–4.6 × 10^7^ protoplasts per gram fresh tissue and 80–95% viability in selected tea cultivars, and tissue culture plant material was found most appropriate for protoplast isolation.

**Conclusions:**

In conclusion, we reported an efficient protocol for isolation of protoplasts from tender tea leaves of all major tea-types (China, Assam and Cambod) grown in India. Moreover, the protocol is also effective for tender-leaf tissue of field-grown, hydroponically-grown and tissue culture-grown tea plants. The findings are expected to contribute to the genetic improvement of tea traits widely.

**Supplementary Information:**

The online version contains supplementary material available at 10.1186/s13007-023-01120-z.

## Introduction

*Camelia sinensis* (L.) O. Kuntze belonging to the Theaceae family is one of the most famous beverages due to its umami flavor and health benefits. About 5100 accessions of tea have been conserved collectively in India and China [1–3] and most of the genetic stocks were directly or indirectly introduced into tea-growing countries from either India or China [4], however, some secondary exchange also occurred between tea producing countries [5, 6]. Tea leaves are rich in polyphenols, which are bioactive compounds responsible for beneficial health effects such as antioxidant, anti-carcinogenic, anti-fungal, anti-viral, anti-aging, thermogenic properties, and help in the treatment of skin diseases, cholesterol reduction, cardiovascular disorders, diabetes, increased metabolism and prevention of tooth decay [7–11]. Therefore, *C. sinensis* leaf beverages have become increasingly popular. However, the market still lacks improved tea varieties with high polyphenol content, enhanced aroma, enhanced theanine content, and improved abiotic and biotic stress tolerance due to obstacles in conventional breeding. The tea genome is highly heterozygous due to its cross-pollinated nature, and its conventional breeding is associated with long breeding cycles, complex offspring, and unstable genetic traits. However, the availability of the genome provides a better opportunity for value addition of the tea but the development of genetically modified tea is still a task as due to the presence of the various secondary metabolites it is difficult to transform and regenerate shoots from tea explants or callus. At least one year possibly is required for the development of the transgenic plants, and additional years are required for further tea transplantation [12]. Moreover, several years are required for experimental work to establish the stability and germline transmission in transgenic plants. Therefore, efficient protocols are required to achieve the improvement in tea traits.

Protoplasts-technology is used as a promising tool to study various aspects of cell biology, genetics, plant physiology, cell division, differentiation, cell wall regeneration, cell ultrastructure, for genetic transformation, and transient transformation, and also has the potential for use in synthetic biology [13–19]. Protoplasts have the ability to regenerate into a whole plant under suitable conditions. Protoplast fusion has been successfully used to develop new varieties by breaking the barrier of conventional breeding which requires compatible parent plants [20–22]. However, an adequate quantity of highly viable protoplast is required for studying/applying these aspects. Isolation of the protoplast from the herbaceous plants has been standardized successfully, like in Arabidopsis [23], rice [24], maize [25], and carrot [18]. Although it is a tough task to isolate protoplast from perennial woody plants still it has been standardized for perennial plants like populous [26], peach [27], apricot [28], citrus [29, 30], Ginkgo biloba [31], Jasminum spp [32], areca palm [33] etc. In the case of *C. sinensis*, Gunasekare and Evans, 1998 [34] reported first the isolation of protoplast from tea and showed that protoplast viability is only dependent on the composition of the enzyme mixture. Afterward, about 19 years later Liu et al., 2017 [35] and Peng et al., 2018 [36] also reported the protocols for protoplast isolation from tea but their efficiency and yield of protoplast were low. Further, Zhou et al., 2021 [37] and Xu et al., 2021 [38] reported the protoplast isolation in tea with enhanced efficiency and yield of protoplast from Chinese tea cultivars. They reported that the protoplast isolation not only depends on the enzyme concentration but also on the type and maturity of the explant and also on the concentration of mannitol used. However, 10–16 h incubation at 25ºC is required to get the protoplast. In 2022, another report by Wang et al., 2022 [39] added snailase enzyme in the protoplast isolation buffer along with cellulase and macerozyme and reduce the incubation time to 4 h for protoplast isolation. However, they shortened the incubation time and enhanced the yield of the protoplast but the viability of the protoplast was lower (70–80%) as compared to previous studies. All earlier developed protocols for the isolation of protoplasts were optimized for tea-types (China and Assam type) and Chinese cultivars grown in China and these existing protocols were found ineffective on Chinese cultivars/different types of tea grown in India due to type/cultivar variability. Therefore in the present study, efforts were made to isolate protoplast from different types of tea including China type, Assam type and Cambod types grown in India. As in earlier studies, different explants like roots, branches, and leaves were used, and in most cases, young actively growing leaves were found suitable for protoplast isolation. Therefore, here we used field-grown, hydroponically grown and tissue cultured-grown plants to standardize the protoplast isolation protocol. We also standardized the mannitol concentration, time and temperature for incubation, shaking conditions, and speed for centrifugation for the isolation of tea protoplasts. The standardized protocol can be used for the improvement of other tea cultivars by using the protoplast system to study the transient expression of genes, to develop foreign DNA-free genome-edited plants through RNP-mediated transformation, protein subcellular localization studies, bimolecular fluorescence complementation assays as well as other in-vivo molecular studies and also in genomics, proteomics and synthetic biology research.

## Materials and methods

### Plant materials

#### Field-grown leaf explants

**Fig. 1:**
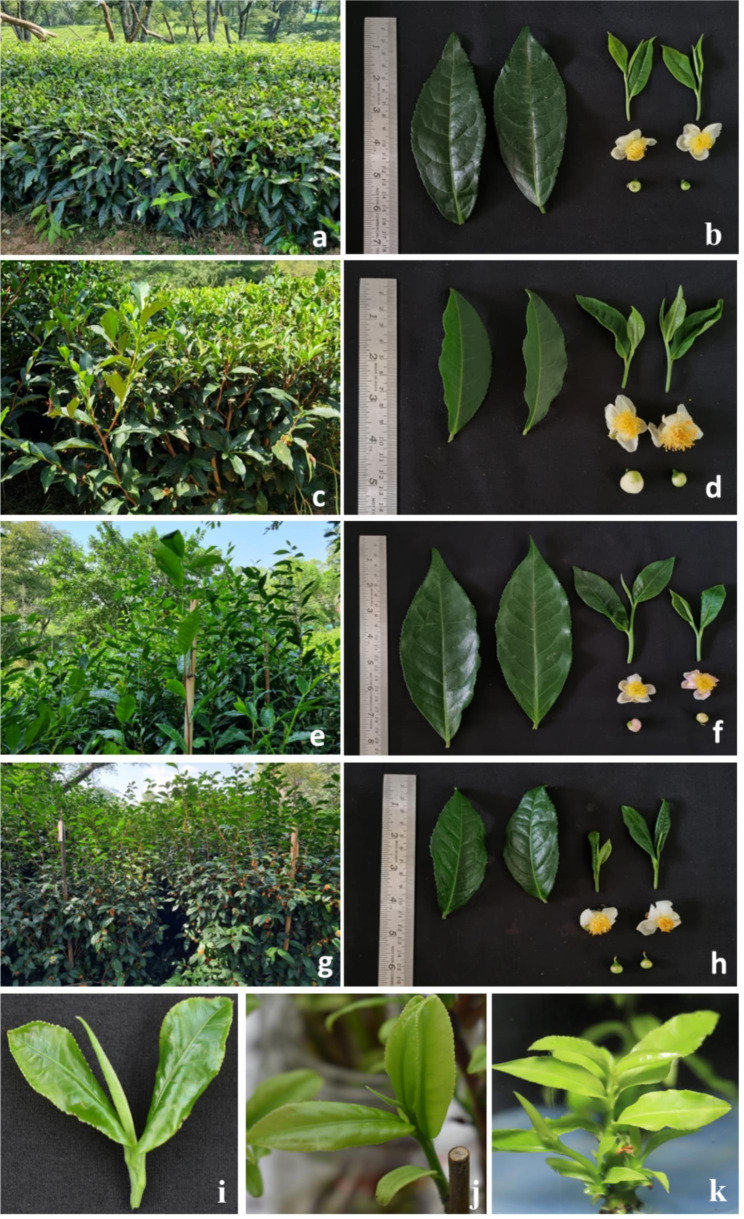
Different tea cultivars used in the study. Tea cultivars (**a**) UPASI-09, (**c**) Him Sphurti, (**e**) TV-23 and (**g**) Kangra Asha growing in the Institute’s Experimental Tea Farm at Banuri, Palampur, Himachal Pradesh, India (1292 m asl, 32.6^o^N and 78.19^o^E). Mature leaves, two leaves and a bud, flower and flower bud of (**b**) UPASI-09, (**d**) Him Sphurti, (**f**) TV-23 and (**h**) Kangra Asha tea cultivar’s. Pictures representing (**i**) Field grown, (**j**) Hydroponically grown, (**k**) Tissue culture grown leaf explants from cv. Kangra Asha (China type)

Four tea cultivars including Him Sphurti (Accession No. IHBT-117, Accession code: CEF-02, China type), TV 23 (Accession No. IHBT-176, Accession code: TV-23, Cambod type), Upasi 9 (Accession No. IHBT-182, Accession code: UPASI-09, Assam type), and Kangra Asha (Accession No. IHBT-122, Accession code: Kangra Asha, China type) belong to three types of tea were used in this study (Fig. 1). First and second leaf explants of twenty-five-year-old bushes of selected tea cultivars growing in the CSIR-Institute of Himalayan Bioresource Technology (CSIR-IHBT) institute’s Experimental Tea Farm at Banuri, Palampur, Himachal Pradesh, India (1292 m asl, 32.6^o^N and 78.19^o^E) were obtained during the seasons of fresh growth (March-May). Distinct morphological features of different tea cultivars used for their identification are supplemented in Table S1 (Fig. 1). The leaf explants were washed and treated with 0.1% sodium hypochlorite for 10 min. The treated leaves were washed three times with distilled water and then used for protoplast isolation.

#### Hydroponically grown leaf explants

One-year-old stem cuttings of about 30 cm were obtained from the above-mentioned tea farm and mature tea leaves were removed without damaging the newly emerging bud. The stem cuttings were washed under running tap water and then washed three times with distilled water containing tween-20 (0.1%). The stem cuttings were then treated with 0.1% sodium hypochlorite for 15 min and again washed with distilled water three times. The treated stem cuttings were placed in the glass jar containing autoclaved distilled water supplemented with 1 mg/l Gibberellic acid (GA3) and placed in a growth chamber at 25ºC. The newly emerged 1st and 2nd leaves (Fig. 1j) obtained after 15 days were used for protoplast isolation.

#### Tissue culture-grown leaves

To establish the in-vitro cultures of selected tea cultivars, the terminal and axillary buds of green stems were taken from the fresh growth of the year (March-May) from the tea orchard (Institute’s Experimental Tea Farm at Banuri, Palampur, Himachal Pradesh, India). First, the buds were washed under running tap water and then washed with distilled water containing tween-20 (0.1%). The buds were then treated with 0.1% sodium hypochlorite for 10 min, and washed with distilled water three times. Then the buds were initially cultured on MS media containing 1 mg/l GA3 for shoot induction. After 35–40 days the induced shoots were cultured on MS media containing 3 mg/l 6-benzylaminopurine (BAP), 0.1 mg/l indole-3-butryic acid (IBA) and 0.5 mg/l Gibberellic acid (GA3). After 6–7 weeks the leaves from multiplied shoots of second cycle of multiplication of tissue culture plants were used for protoplast isolation (Fig. 1k). The tissue culture leaves were multiplied and maintained in a tissue culture chamber with 25 ± 2˚C temperature and 16 h/8 h light-dark cycle respectively.

### Preparation of the enzyme solution

Fresh enzyme solutions were prepared before the isolation of the protoplasts. To standardize the protoplast isolation for selected tea cultivars, three enzymes 1–3% cellulase R-10 (Duchefa Biochemie), 1% hemicellulase and 0.2-1% macerozyme R-10 (Duchefa Biochemie) were used. The enzymes were dissolved in a protoplast salt solution (modified from Xu et al., 2021) containing 20 mM 2-ethanesulfonic acid (pH 5.7), 0.6 M mannitol, 10 mM CaCl_2_, 20 mM KCl and 0.1% bovine serum albumin. The 4% polyvinylpyrrolidone (PVP) was also added to prevent the oxidation of the phenols, as oxidized phenol may interfere with protoplast isolation. The different mannitol concentrations were tested in the range of 0.2-1.0 M for optimum osmotic pressure to enhance the integrity of the isolated protoplast. The solution containing the enzymes was heated at 55ºC for 15 min to dissolve and activate the enzymes. The enzyme solution was cooled down to room temperature and then filtered through 0.22µ syringe filters. The filter-sterilized enzyme solution was further used for the hydrolysis of leaf tissue.

### Isolation of the protoplast

**Fig. 2:**
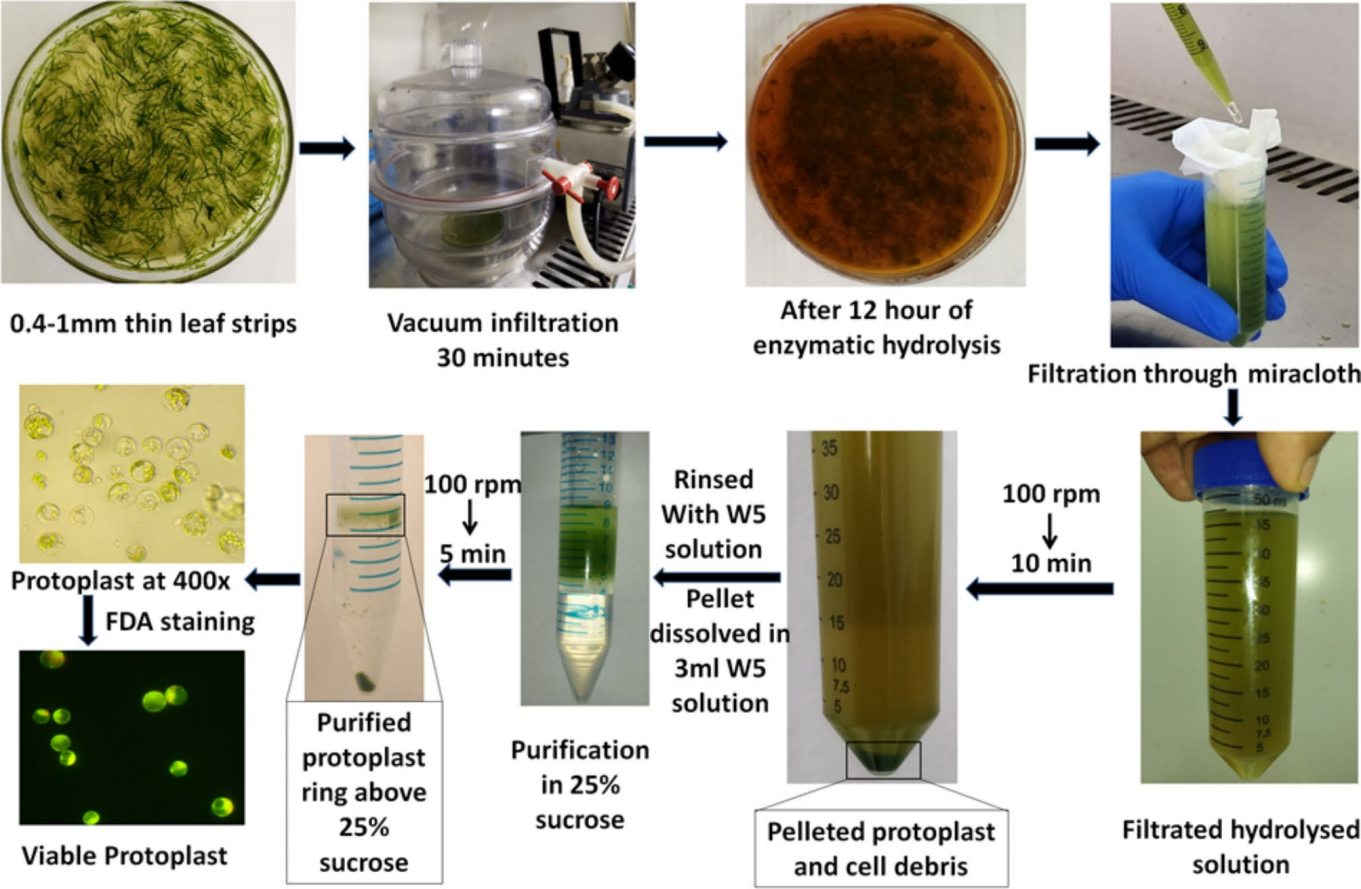
Standardised procedure for isolation of protoplast from tea leaf explants

Three types of leaf explants obtained from field-grown, hydroponically-grown and tissue culture-grown tea cuttings were used for protoplast isolation. The excised leaf explants were placed in sterile distilled water before cutting into fine sections. The main ribs and leaf margins were removed and then about 1–2 g leaf explants were cut into fine strips (0.5-1.0 mm) using the sharp blade. This process takes time therefore tissue strips were placed in 0.6 M mannitol solution during this process to maintain the osmotic balance. When whole tissue was excised into fine strips, the mannitol solution was discarded, and 25 ml enzymatic solution was added to the tissues. A negative pressure of 400 mm Hg was applied to enhance the infiltration of the enzymes. The tissue was then incubated at 25–28 ºC with a gentle shaking of 60 rpm for lysis of tissue and release of the protoplast into the osmotic solution. The used enzymes are light sensitive therefore the whole process was done under dark conditions. The detailed methodology for protoplast isolation is given in Fig. 2. The major factors that influence protoplast isolation like concentration of cellulase R-10 (1–3%), macerozyme (0.2-1.0%), mannitol (0.2-1.0 M), time for vacuum infiltration (10–40 min) and digestion time (8–16 h) were tested to standardized the protocol. The different types of leaf explants obtained from field-grown, hydroponically-grown and tissue culture-grown tea plants were also tested and compared for protocol efficiency and yield of protoplast.

### Protoplast purification

Once the enzymatic hydrolysis process was completed, protoplasts were first purified by coarse filtration through Mira cloth (EMD Millipore Corp.) and then centrifuged at 100 g for 10 min. The upper phase was discarded carefully without disturbing the pelleted protoplast. The pelleted protoplasts were resuspended with gentle swirling in W5 solution [2 mM MES, 154 mM NaCl, 5 mM KCl, 125 mM CaCl_2_; pH 5.7 [38]. The resuspended filtrate was centrifuged at 100 rpm for 5 min to settle down the protoplast. The upper phase was discarded and the pellet was resuspended in 3 ml of W5 solution. The 25% sucrose was used for the purification of protoplast. The 7 ml 25% sucrose solution was poured into a 15 ml centrifuge tube and then 3 ml protoplast suspension was overlayered carefully using 1 ml micro tip excised at the bottom using a scissor and centrifuged at 100 rpm for 5 min. The purified protoplast appeared as a green layer above the sucrose solution, which was collected and stored for a short period on ice for further use.

### Protoplast yield and viability test

**Fig. 3:**
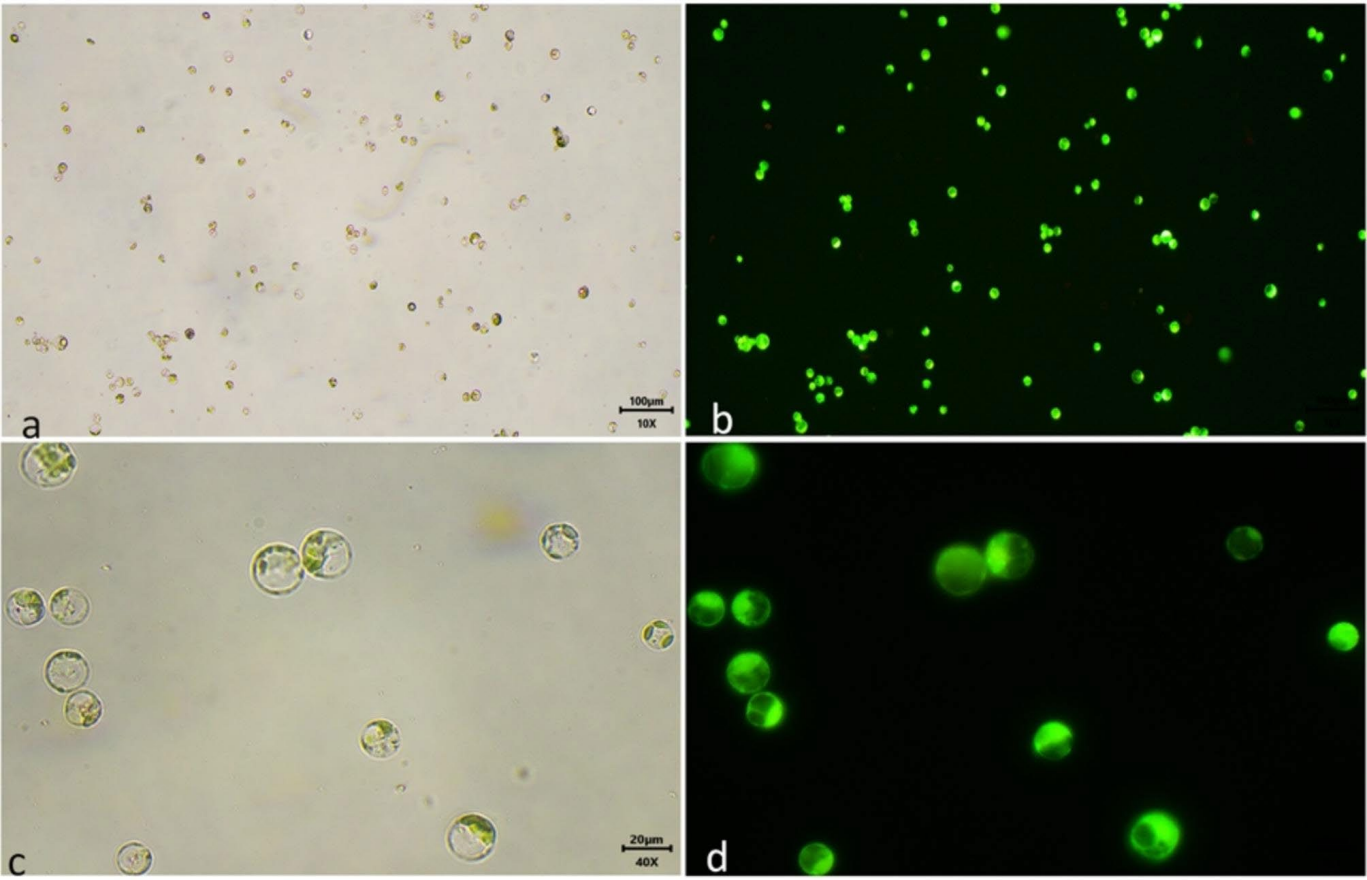
Viability testing using FDA stain. (**a**) Under bright light at 100x (bar-100 μm), (**b**) Under 480 nm excitation at 100x (fluorescence-labelled viable protoplasts), (**c**) Under bright light at 400x (bar-100 μm) and (**d**) Under 480 nm excitation at 400x(fluorescence-labelled viable protoplasts)

A hemocytometer was used to determine the protoplast yield using a fluorescence microscope (Magnus MLXi Plus) under bright light. The protoplast yield was calculated as how many numbers of protoplasts were yielded per gram of leaf tissue. To determine the viability of protoplast 0.01% (W/V) FDA stain was used as per protocol [33]. The protoplasts were observed after staining with FDA under a fluorescence microscope (Magnus MLXi Plus). The protoplasts which were visible as green under excitation of 480 nm are viable. The protoplast viability is calculated in terms of percentage i.e. how many protoplasts are visible green from the total protoplast under view (Fig. 3). Viability was calculated for each sample under at least three fields view and each experiment was performed in replication of three.

### Statistical analysis

IBM SPSS Statistics for Windows, version 20 (IBM Corp., Armonk, NY, USA) was used for the analysis of the data. Differences between treatments were considered significant at P ≤ 0.05 according to the least significant difference. Data are represented as means ± standard errors of the mean from three independent experiments.

## Results and discussion

### Optimized procedure for protoplast isolation

China is the largest and India is the second largest producer of tea. In addition, China is the largest producer of green tea and India is the largest producer of black tea (FAO/EST 2022). Earlier developed protocols for the isolation of tea protoplasts were standardized only for Chinese cultivars grown in China [35–39]. The origin of the tea plant is believed from Yunnan Province in southwestern China, and China has the largest plantation, production and consumption of tea in the world [3], which may be the possible reason for the development of earlier optimized protocols on only Chinese cultivars. Zhou et al., 2021 [37] identified *C. sinensis* var. *sinensis* cv. Zhongbai 4 as the most suitable line for mesophyll protoplast isolation. Isolation of protoplast from different tissues of hydroponic cutting seedlings, tea plantation seedlings and potted seed seedlings of var. sinensis and var. assamica of four Chinese cultivars (Shuchanzao, Zijuan, Huangkui and Huangshanbaicha) was performed by Xu et al., 2021 [38]. Recently, Wang et al., 2022 [39] could establish a fast protoplast isolation method which was also specific to *C*. *sinensis* var. *sinensis* cv. Shuchazao. Therefore, earlier optimized protoplast isolation protocols were based on Chinese cultivars including var. sinensis (China type) and var. assamica (Assam type) tea types only. As all major three types of tea (China, Assam and Cambod types) have distinct compositions of secondary metabolites, therefore have distinct importance and are commercially used. Hence efficient protoplast isolation protocol is required for all types of tea. Huge variation in secondary metabolites in different tea types and cultivars limited the successful application of various approaches used for its trait improvement. The efficiency of a protocol for the isolation of protoplast is specific to the types and cultivars of tea plants. Source material for isolation of protoplast is considered as an important factor for successful isolation of protoplast, and protoplast yield was found to vary with different tea cultivars which might be due to the variation in content of polyphenol, cellulose and pectin in leaf tissue which inhibit the enzymatic digestion resulting poor protoplast yield [31, 37]. Preliminary studies in our laboratory showed that the earlier reported protocols [which were optimized for tea-types (China and Assam type) and Chinese cultivars grown in China] could not efficiently work on the important tea types and cultivars grown in India might be due to type/cultivar variability. As Indian-tea production has specific importance as the second largest-tea producer, therefore, a protoplast isolation procedure for cultivars grown in India needs to be established. Therefore, in the present study efforts were made to optimize the protocol for the isolation of protoplast from all types of tea (i.e. China, Assam and cambod type) in selected cultivars grown in India. Keeping in view the above points we standardized protocols for four tea cultivars i.e. Him Sphurti (China type), TV 23 (Cambod type), Upasi 9 (Assam type), and Kangra Asha (China type) belonging to three types of tea.

After 12 h of digestion of the tissue at 28ºC and 60 rpm in an incubator shaker, hydrolyzed tissue was passed through the Mira cloth membrane. The filtrate was centrifuged at 100 x g for 10 min and then pelleted protoplast and debris were washed with 10 ml W5 solution at 100 x g for 5 min. The step was repeated twice to remove the debris material. The pelleted protoplast was resuspended in 3 ml W5 buffer and poured above the 6 ml 25% sucrose solution and centrifuged at 100 x g for 5 min. The purified protoplast remains above the sucrose solution and can be collected with 1 ml microtip excised with scissors at the bottom. The purification procedure was carried out at 4ºC to enhance the viability of the protoplast.

### Effect of enzyme concentration on protoplast isolation

**Fig. 4:**
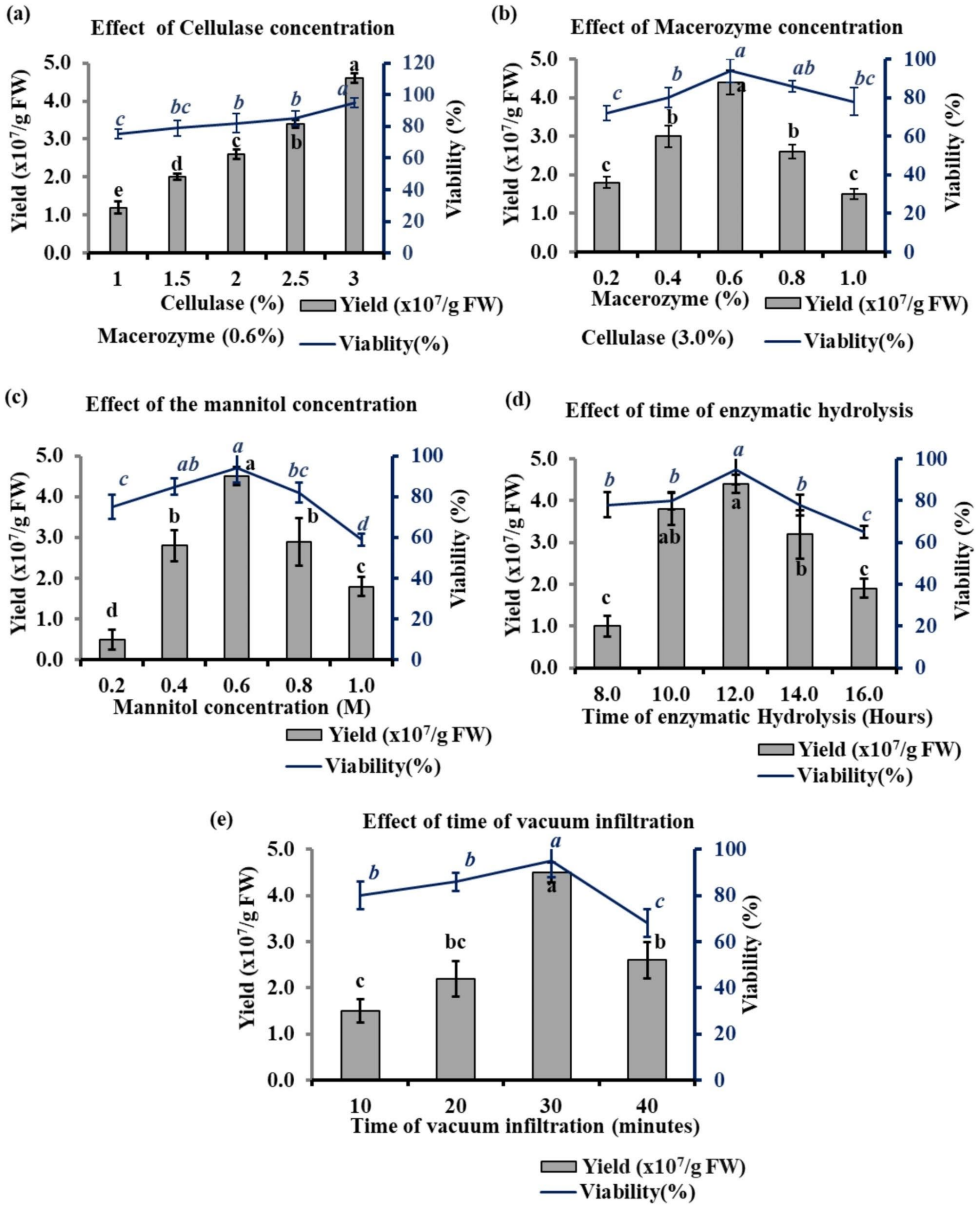
Effect of cellulase concentration (**a**), macerozyme concentration (**b**), mannitol concentration (**c**), time of enzymatic hydrolysis (**d**) and time of vacuum infiltration (**e**) on protoplast yield and viability. These optimization were performed using tea cv. Kangra Asha. Different small letters represent statistically significant differences at *P* < 0.05

The enzyme concentration is most critical for the isolation of protoplast. To find out the optimal enzyme concentration for protoplast isolation 1st and 2nd tender leaves of all selected tea cultivars were digested with enzymes for 12 h duration at 28ºC, 60 rpm. The result of different concentrations and combinations of the enzymes were presented in Fig. 4. The yield of protoplast enhanced with the increase in the concentration of cellulase. The lowest yield of 1.2 × 10^7^/g FW (gram Fresh weight) was observed on 1% cellulase along with 0.6% macerozyme and 1% hemicellulase. However, the highest yield of 4.6 × 10^7^/g FW with 95% viability was observed when 3% cellulase was used (Fig. 4a). Although, the viability of the protoplast varied non-significantly among different concentrations of cellulase used. In the case of macerozyme, the yield of protoplast increased initially but with the increase in concentration fell after reaching the optimum value. The highest yield of 4.4 × 10^7^/g FW with 94% viability was observed on 0.6% macerozyme along with 3% cellulase and 1% hemicellulase (Fig. 4b). However, the yield fell to 1.5 × 10^7^/g FW with 78% viability when 1% macerozyme was used, therefore 3% cellulase along with 0.6% macerozyme and 1% hemicellulase was considered best for the isolation of the protoplast from tender tea leaves.

### Effect of mannitol concentration on yield and viability of protoplast

Mannitol was used as an osmotic pressure regulator in the current study. The effect of different mannitol concentrations was tested on the yield and viability of protoplast. It was observed that protoplast yield and viability increased initially with the increase in mannitol concentration and then started to decrease at higher concentrations. At a lower concentration (0.4 M) of mannitol, the protoplast yield was very low and the lower number of protoplast remains viable. The 0.6 M mannitol concentration was found optimum for protoplast isolation when cellulase R-10 concentration was 3% and macerozyme R-10 concentration was 0.6% and hydrolysis was done for 12 h at 28ºC, 60 rpm. The yield at this concentration was 4.5 × 10^7^/g FW and viability were 94% (Fig. 4c). When the concentration was enhanced to 0.8 M and 1.0 M, a low number of viable protoplast was obtained and more burst and deformed protoplasts were obtained. Therefore 0.6 M mannitol concentration was found optimum for protoplast isolation.

### Effect of time of enzymatic hydrolysis

Enzymatic hydrolysis of tea leaf strips with 3% cellulase, 0.6% macerozyme and 1% hemicellulase was tested for different time durations of 8, 10, 12, 14, and 16 h. The yield of the protoplast seems to increase with the increase of digestion time initially. The lowest yield of 1 × 10^7^/g FW was obtained at 8 h and the highest protoplast yield of 4.4x x10^7^/g FW was observed after 12 h of digestion (Fig. 4d). The yield starts to decrease after 12 h of digestion and at 16 h yielded only 1.9 × 10^7^/g FW protoplasts. However, hydrolysis time did not have a significant impact on viability but at 16-hour digestion time low viability of 65% was observed, and more burst and distorted protoplast was visible under the microscope.

### Effect of vacuum treatment on protoplast isolation

The vacuum pre-treatment before incubation for hydrolysis by enzymes is found to be an important step for protoplast isolation. The vacuum treatment enhances the infiltration of the enzyme inside the leaf tissue and increases the release of the protoplast. The excised leaf strips of 1 mm dipped in enzyme solution were treated with 400 mmHg vacuum pressure for different time durations. It was observed that the yield of the protoplast was increased with vacuum treatment and protoplast with morphology comparable to untreated tissue and less cell debris were obtained. The vacuum treatment for a certain range of time seems to enhance the yield and viability percentage of the protoplast. When a vacuum pressure of 400 mmHg was applied for 30 min, a maximum protoplast yield of 4.5 × 10^7^/g FW was obtained with 95% viable protoplast (Fig. 4e). However, when the vacuum pressure was enhanced to 40 min less number of viable protoplast were obtained and more deformed or bursted protoplast were observed. Therefore, 30 min of vacuum treatment at 400 mmHg was found optimum for protoplast isolation from selected tea cultivars.

### Effect of type of leaf explant on protoplast yield and viability

**Fig. 5:**
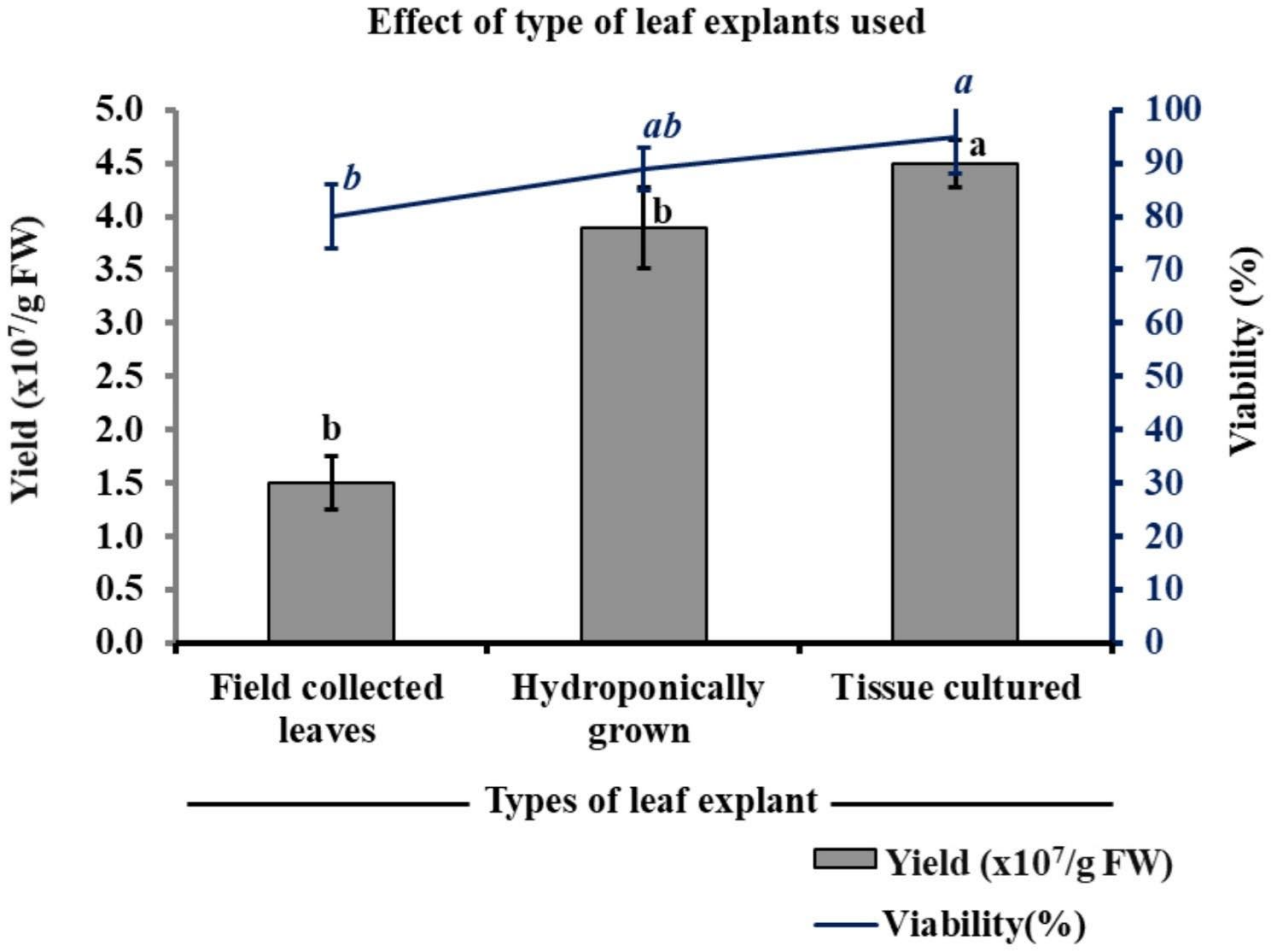
Effect of types of leaf explants on yield and viability of the protoplast. Different small letters represent statistically significant differences at P < 0.05

**Fig. 6:**
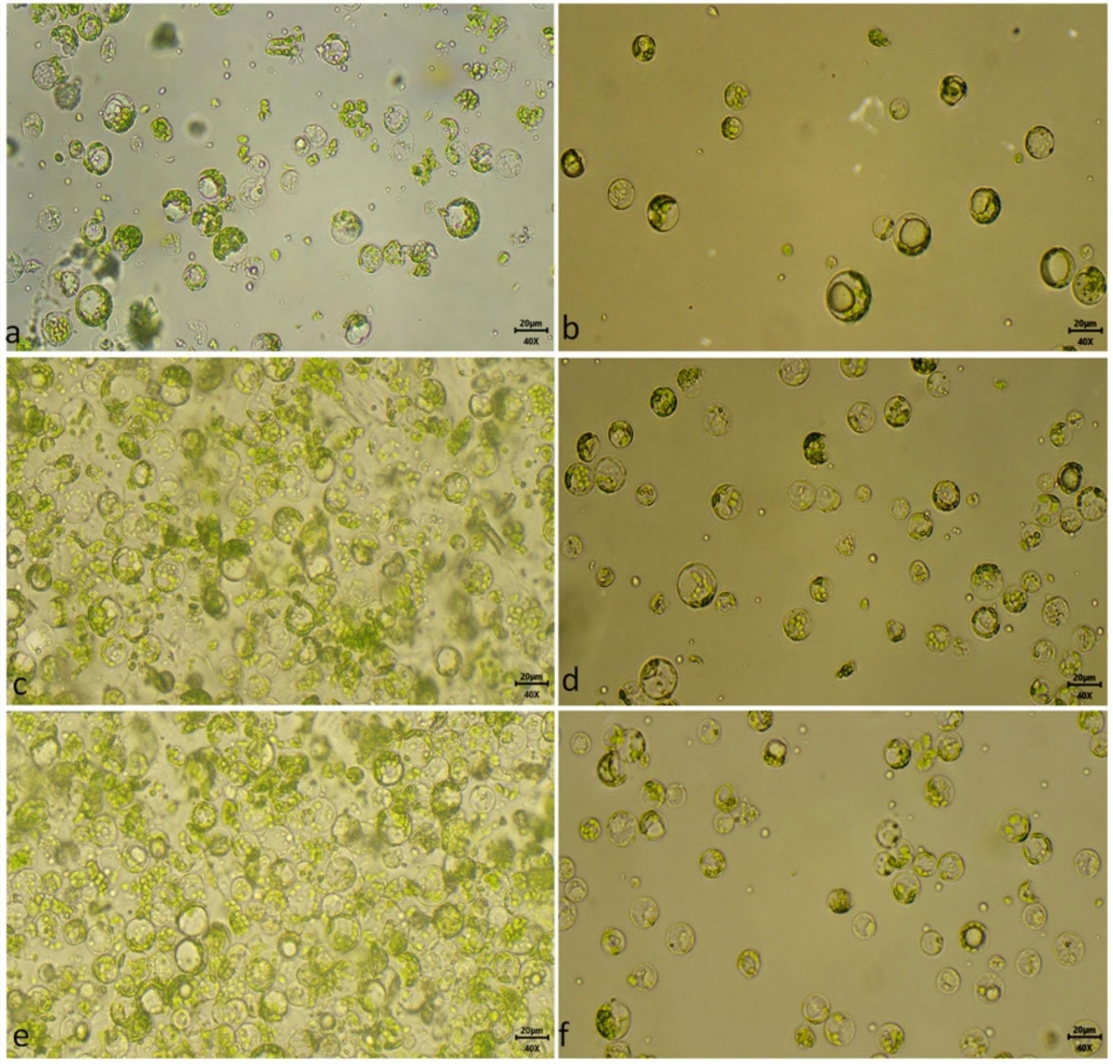
Effect of type of leaf explant on protoplast isolation before and after purification with 25% sucrose (**a, b**) field-grown, (**c, d**) hydroponically grown, and (**e, f**) tissue culture grown leaf explants, respectively. (400x and bar = 20 μm)

The effect of different types of leaf tissue (from field-grown, hydroponically-grown and tissue culture multiplied) on protoplast isolation was studied. The type of leaf tissue had a significant impact on the protoplast yield and viability (Fig. 5). Only 1st and 2nd leaves were used in the current study, as in most previous studies only these leaves showed good results. The lowest yield of 1.5 × 10^7^/g FW with 80% viability of the protoplast was observed in the field-grown leaves (Fig. 5). More phenolic compounds in the field-grown leaves might interfere with protoplast isolation. However, in the case of hydroponically grown leaf explant, an enhanced protoplast yield of 3.9 × 10^7^/g FW with 89% protoplast viability was observed. But the best results were observed from the in-vitro tissue culture-grown leaf explants, which resulted in a yield of 4.5 × 10^7^/g FW with 95% viability (Fig. 5). Therefore, in-vitro tissue cultured multiplied shoot leaves were found to be best for protoplast isolation, although hydroponically grown shoots also showed comparable results and could also be used for protoplast isolation (Fig. 6).

### Effect of different cultivars on protoplast yield and viability

**Fig. 7:**
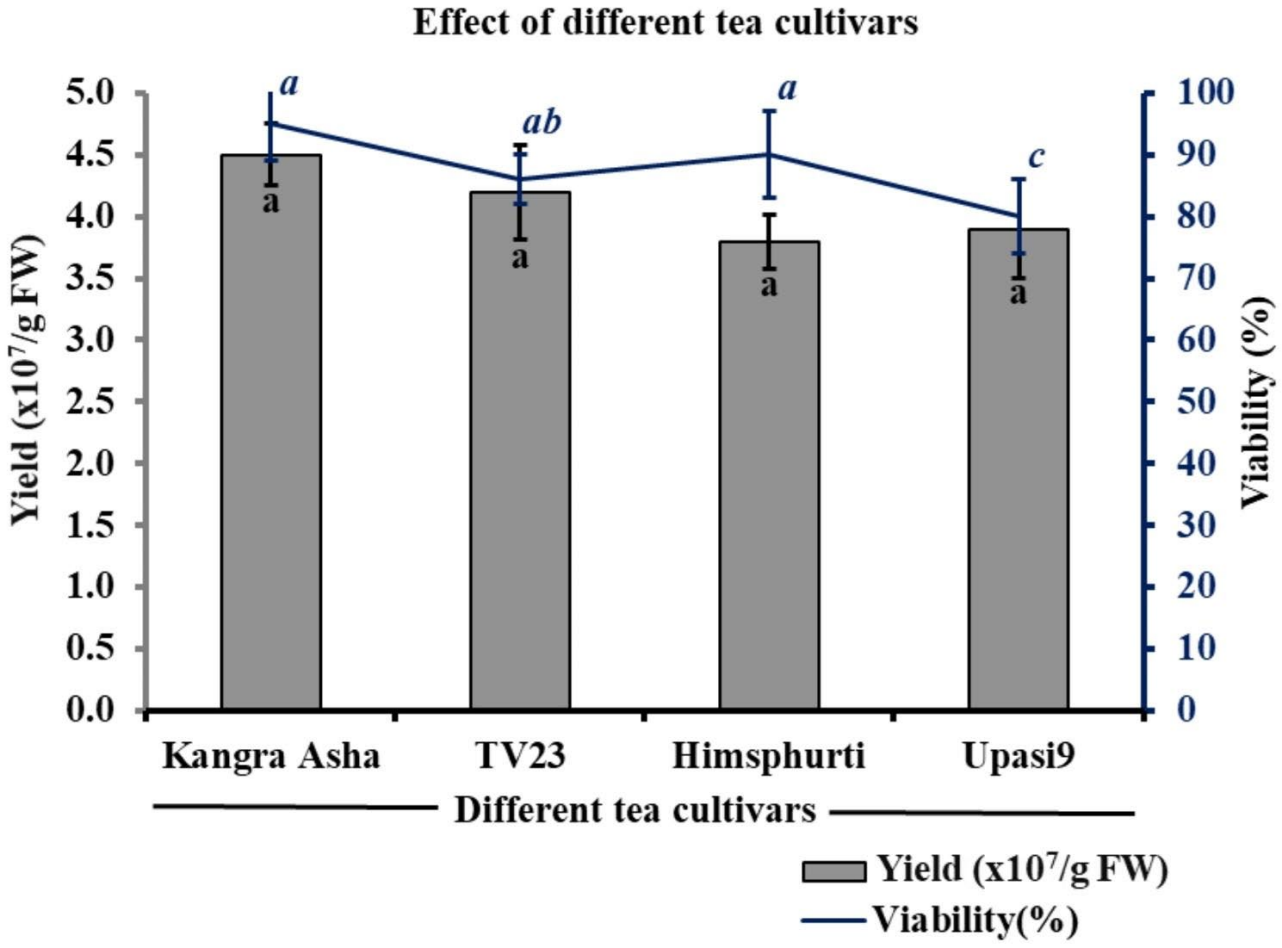
Effect of different tea cultivars on protoplast yield and viability. Different small letters represent statistically significant differences at *P* < 0.05

**Fig. 8:**
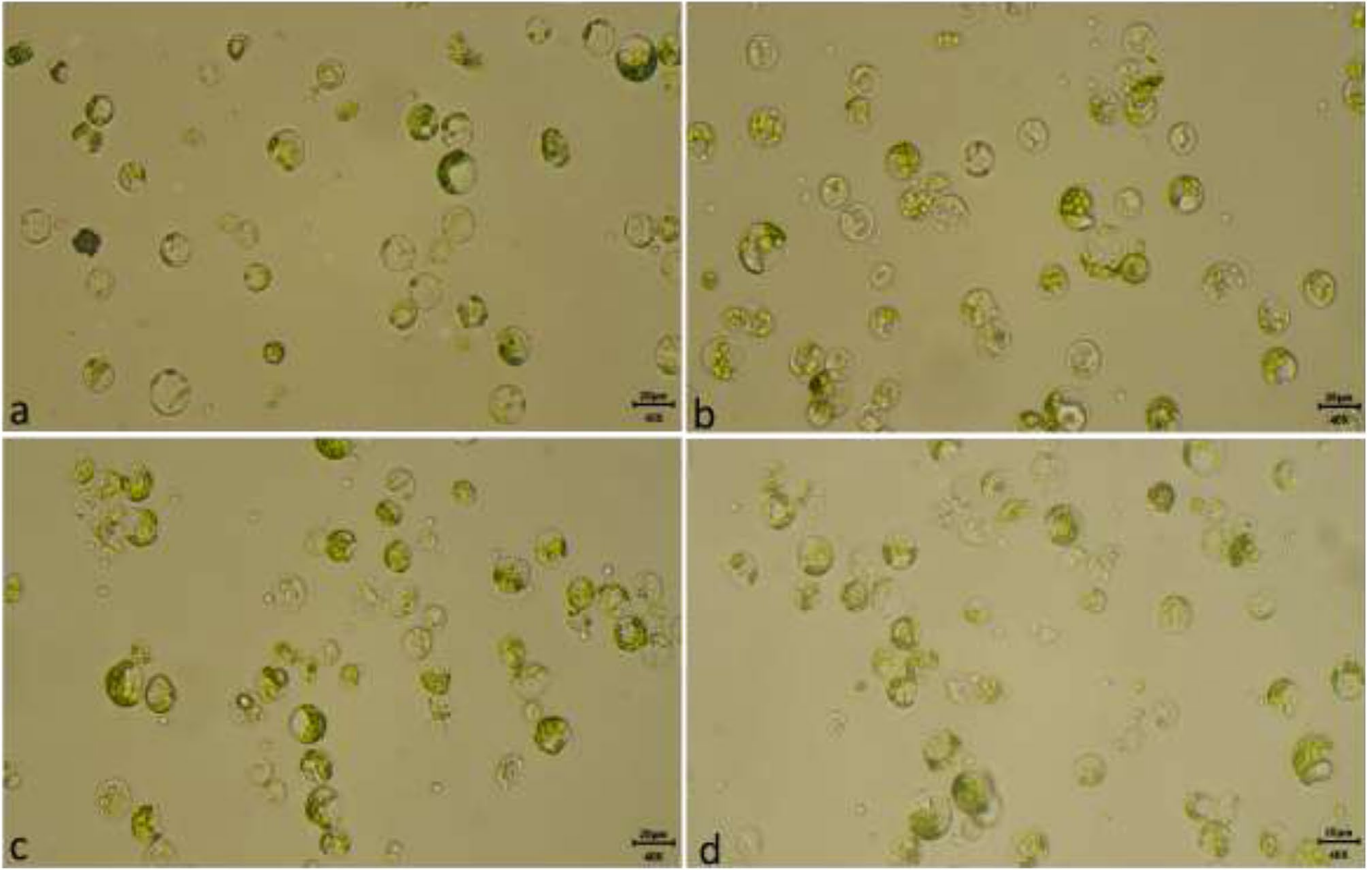
Effect of different tea cultivars on protoplast isolation. (**a**) TV23 (**b**) Kangra Asha (**c**) Him Sphurti and (**d**) Upasi 9 (at 400x and bar = 20 μm)

The protocol worked well for all four selected tea cultivars (Him Sphurti, TV 23, Upasi 9, and Kangra Asha), and protoplast yield and viability did not vary significantly among these cultivars. Although, the Kangra Asha cultivar was a little more responsive for protoplast preparation and yielded 4.5 × 10^7^/g FW protoplast with 95% viability (Fig. 7). The yield of TV23, Him Sphurti and Upasi 9 was 4.2 × 10^7^/g FW, 3.8 × 10^7^/g FW, 3.9 × 10^7^/g FW, respectively with 80–90% viability (Fig. 8). Therefore, this protocol may work well for the isolation of protoplast from most cultivars and types of tea grown in India.

**Fig. 9:**
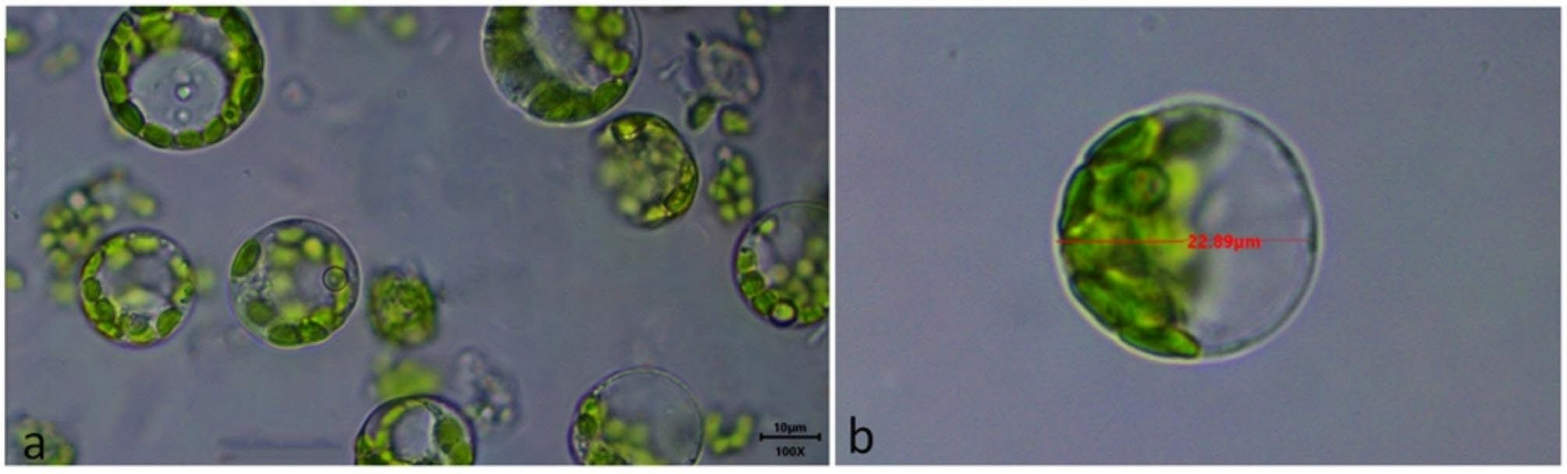
(**a**) Purified protoplast at 1000x (bar = 10 μm). (**b**) Protoplast size measured using scale bar at 1000x

Protoplasts were obtained more efficiently from the tissue culture and hydroponically-grown leaf explants. The yield and viability were also significantly higher among these tissues as compared to field-grown leaf explants. This may be because of the tenderness of the tissue as it seems to be a key factor for the preparation of protoplast from tea plants, as reported in previous studies on perennial plants [33, 38–40]. However, in some studies, it was reported that tissue culture explants were better than hydroponically grown explants [41] but in our studies, these two showed comparable results. But the tissue cultured grown explants are more tender in nature and grown in a controlled environment, moreover available throughout the year for protoplast isolation. Therefore, they might be better candidates for protoplast isolation. Overall the protoplast isolated from tea leaf explants of field-grown, hydroponically-grown and tissue cultured-grown leaf explant is ~ 23 μm in diameter and viable protoplast appear round in shape with mostly enriched with chloroplast in all four cultivars (Fig. 9). Such an efficient protocol for protoplast isolation from major three types of tea with such high yield and viability is reported for the first time in tea cultivars grown in India.

Previous studies on protoplast isolation from tea were only based on the Chinese cultivars grown in China. Earlier, the yield of the protoplast was enhanced from 4.1 × 10^6^/g FW [as obtained in 1st report on tea protoplast isolation [34], to 6.6 × 10^6^/g FW in one of the recent studies by Wang et al., 2022 [39], where the snailase was added which could shorten the time of protoplast isolation from 16 h to 4 h but the viability was also lowered (73–82%). Earlier the higher cell viability (92.94%) was obtained but the protoplast yield was 3.27 × 10^6^/g FW [38]. Moreover, the addition of snailase also adds extra cost to the protoplast isolation protocol. The study by Xu et al., 2021 [38] reported the isolation and purification of protoplast from two types of tea (China and Assamica) including four cultivars (Zijuan, Shuchazao, Huang shanbaicha and Huangkui). Xu et al., 2021 used various tissues, including the roots, branches, leaves, and also proposed tender leaves as a best material for protoplast isolation. They could achieve the best protoplast yield of 3.27 × 10^6^ g^− 1^ FW and 92.94% viability using tender leaves of China type tea cv. Shuchanzao, and reported 1.5% (w/v) cellulase and 0.4–0.6% (w/v) macerozyme in a solution containing 0.4 M mannitol, enzymatic hydrolysis over 10 h, and an iodixanol concentration of 65% as the optimal conditions for protoplast isolation and purification. We found that the optimum conditions reported by Xu et al., 2021 was ineffective in tea cultivars grown in India. Therefore, here, efforts were made to develop an efficient protoplast-isolation protocol from all major tea-types (China, Assam and Cambod types) grown in India and also from three types of tender leaves obtained from field-grown, hydroponically-grown and tissue culture-grown tea plants. In the present study enhanced yield of 4.5 × 10^7^/g FW was obtained from the tissue-cultured leaves, which may be due to the tenderness of the leaves and lesser phenolic compounds. The other key factors like temperature modified from 25 to 28 ºC, addition of hemicellulase (1%), rpm of the centrifugation (100 rpm) and addition of 4% PVP were standardized during the establishment of the protocol; therefore these factors also play critical roles in the enhancement and viability of the protoplasts of selected tea types and needs to be further considered for optimization of protoplast-isolation protocols for unexplored tea cultivars. The developed method in this study could also provide an increased protoplast yield (3.8-4.5 × 10^7^ protoplasts per gram fresh tissue) and viability (80–95%) compared to the earlier report [38]. However, the incubation time for enzymatic hydrolysis was higher (12 h) in the present protocol compared to the earlier report (10 h) [38]. As yet, over 5,100 accessions of different tea germplasm are conserved in China and India [1–3] and the tea-genetic stocks used by other tea-growing countries are also introduced from China and India [4]. Besides, the unique flavor of Chinese and Indian tea also make their quality improvement essential. Therefore, both the Chinese and Indian tea-cultivars have their specific significance. Hence, the development of protocols for both the Chinese and Indian tea-cultivars has specific importance for tea improvement and needs to be done. The protocol established in the current study can be efficiently utilized to isolate protoplast from different types and tea cultivars (especially for cultivars grown in India) with enhanced yield and viability (See Table 1).

**Table 1:** Comparative analysis of protoplast isolation protocol of different woody plants as per literature

Plant	Sample	Enzyme concentration	Incubation conditions (duration, temperature, shaking)		Purification	Yield (protoplast/gFW)	Viability (%)	Source
*Jasminum sambac* and *J. mesnyi*	Callus	1.5% cellulase, 0.4% macerozyme, 0.8% pectinase	4 h	26–30 °C in dark, 50 rpm	21% sucrose, 100×g for 3 min at 4 °C	23.8 ± 4.3 × 10^6^	88	[32]
	Leaves		4 h			5.6 ± 3.9 × 10^6^	89.6	
	Flowers		10 h			3.9 ± 5.0 × 10^6^	83	
	Stems		6 h			3.3 ± 0.5 × 10^6^	72.5	
Holm Oak (*Quercus ilex* L.)	Leaf tissue	2% cellulase, 1% macerozyme	25 °C for 4 h in dark, 50 rpm		11% mannitol, 1000×g for 5 min at RT	61.5 ± 9.7 × 10^6^	-	[42]
Rubber tree	5–7 day old etiolated leaves	1.5% cellulase, 0.6% macerozyme	4–5 h at 26–28 °C in dark, 60 rpm		900 rpm for 3 min at 4 °C	-	-	[43]
Peach	Mesocarp tissue (Fruits)	2% cellulase and 0.2% macerozyme	30 °C for 4 h		300×g for 10 min at 4 °C	-	-	[27]
Asian white birch	Young microculture	0.5% cellulase, 0.1% maceroenzyme	16-18 h, 50 rpm		350×g for 10 min	100 ± 0.3 × 10^4^	95	[44]
‘Boule de Neige’ Rhododendron						260.0 ± 0.3 × 10^4^	99	
‘Gibraltar’ Azalea		2% cellulase, 0.5% maceroenzyme	4–6 h, 50 rpm			52 ± 0.6 × 10^4^	62	
Apricot	Leaves from in vitro shoots	1% cellulase, 0.1% pectolyase, 1% hemicellulase	13–16 h		21% sucrose 75×g at RT	20.97 × 10^6^ (Plasmolysis of the leaves in a 13% sorbitol solution for 90 min)	83	[28]
*Magnolia*	Young leaves	3% cellulase, 0.8% macerozyme, 0.04% pectinase	6 h, 25 °C, 60 rpm		100×g for 10 min	1.89 × 10^5^	-	[45]
*Ginkgo biloba L.*	Leaves	2% cellulase, 0.2% pectolyase, 1.5% macerozyme	5 h in dark at 25 °C, 50 rpm		50×g for 3 min	5.39 × 10^6^	80.23	[31]
*Albizia julibrissin*	Leaves from in-vitro seedlings	1.5% cellulase, 1% pectolyase	6 h in dark at 25 ± 2 °C, 40 rpm		100×g for 5 min	6.31 × 10^5^	87	[46]
	Callus	2% cellulase, 1% pectolyase	16 h in dark at 25 ± 2 °C, 40 rpm			5.53 × 10^5^	85	
*Platycladus orientalis*	Young and fresh scale leaves	1.5% cellulase, 0.4% macerozyme, 0.4% pectolyase, 1.0% ligninase	16 h in dark at 25 °C, 40 rpm		-	9.60 × 10^3^	52.4	[47]
*Areca catechu*	Leaf peels	2% cellulase, 0.5% macerozyme	12 h dark at 25 ℃, 40 rpm		100×g for 3 min	2.5 × 10^7^	86.6	[33]
*Camellia oleifera*	Leaves	1.5% cellulase, 0.5% macerozyme, 0.25% Snailase	28 ℃ in dark, 40 rpm		15×g for 4 min	3.5 × 10^7^	90.9%	[40]
*C. sinensis var. sinensis* cv. ‘shuchazao’	Leaf	1.5% cellulase, 0.5% macerozyme, 0.7% snailase	25 °C for 4 h in the dark		100×g for 2 min with a swinging bucket rotor	3.5–6.6 × 10^6^	73-82%	[39]
*Camellia sinensis* (L.) O. Kuntze Cultivars used: Zijuan, Shuchazao, Huang shanbaicha and Huangkui	Tender leaves	1.5% cellulase, 0.4–0.6% macerozyme	10 h		65% iodixanol 200×g for 3 min	3.27 × 10^6^	92.94	[38]
	Mature leaves					1.48 × 10^6^	83.23	
	Unlignified branches					1.20 × 10^6^	80.97	
						3.20 × 10^6^	89	
	Roots							
*Camellia sinensis* (L.) O. Kuntze Cultivars used: Him Sphurti (China type), TV 23 (Cambod type), Upasi 9 (Assam type), and Kangra Asha (China type)	Tissue cultured	0.6% macerozyme, 3% cellulase, 1% hemicellulase	12 h in dark at 28 ºC, 60 rpm		25% sucrose 100×g for 5 min with a swinging bucket rotor	4.5 × 10^7^	95%	This study
	Field grown					1.5 × 10^7^	80%	
	Hydroponically grown					3.9 × 10^7^	89%	

RT- room temperature

## Conclusion

In the current study, we standardized an efficient protocol for isolation of protoplasts from selected cultivars belongs to all major types of tea (China, Assam and Cambod types) grown in India and also from three types of tender leaves obtained from field-grown, hydroponically-grown and tissue culture-grown tea plants. For developing this high-efficiency protocol, key factors like enzyme concentration, time of enzymatic hydrolysis, mannitol concentration, time of vacuum infiltration, rpm of the centrifugation, and temperature during hydrolysis were studied. The effect of the types of leaf explants and cultivation methods used for growing leaves was also studied. In our results tissue culture-grown leaves were found best for protoplast isolation, however, hydroponically grown 1st and 2nd leaves also showed comparable results. The protocol worked well for all four selected cultivars, therefore this protocol may be utilized for other tea cultivars’ protoplast isolation and would be helpful in the genetic improvement of tea widely.

### Electronic supplementary material

Below is the link to the electronic supplementary material.

Supplementary Material 1: Important features to distinguish different tea cultivars used in the study

## Data Availability

The datasets supporting the conclusions of this article are included within the article.
