# Correction: A highly efficient protocol for isolation of protoplast from China, Assam and Cambod types of tea plants [Camellia sinensis (L.) O. Kuntze]

**DOI:** 10.1186/s13007-024-01176-5

**Published:** 2024-04-24

**Authors:** Abhishek Kumar, Nikhil Rawat, Shweta Thakur, Rohit Joshi, Shiv Shanker Pandey

**Affiliations:** 1 Biotechnology Division, Council of Scientific and Industrial Research (CSIR, Institute of Himalayan Bioresource Technology, Palampur, 176061 India; 2 Academy of Scientific and Innovative Research (AcSIR), Ghaziabad, 201002 India

**Correction: Plant Methods (2023) 19:147**

10.1186/s13007-023-01120-z

In this article the wrong figure appeared as Fig. 8. Figure 8a and d are incorrectly placed (Fig. 8a and d have the same image) and it is given below,
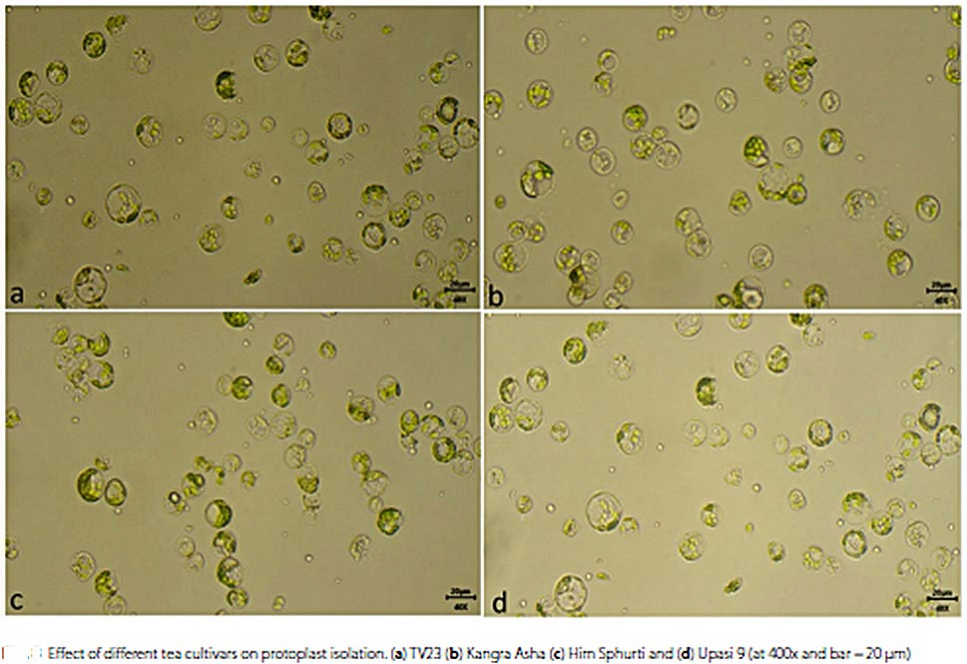


The figure should have appeared as shown below.

**Fig. 8:**
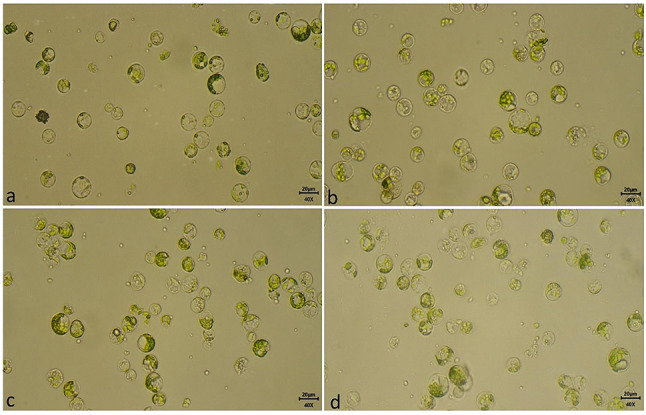
Effect of different tea cultivars on protoplast isolation. (**a**) TV23 (**b**) Kangra Asha (**c**) Him Sphurti and (**d**) Upasi 9 (at 400x and bar = 20 μm)

In this article the statement in the Funding information section was incorrectly given as “The Council of Scientific and Industrial Research (CSIR), India [MLP-0170 (FBR-Genome-Editing Network Project MLP-008)] financially supported this study.” and should have read “The Council of Scientific and Industrial Research (CSIR), India [MLP-0170 (FBR-Genome-Editing Network Project MLP-007)] financially supported this study.”

In the Acknowledgements section, additional sentence has been included “This manuscript represents CSIR-IHBT communication number 5460.’’.

The original article has been corrected.

