# Supplementary material for: A highly efficient protocol for isolation of protoplast from China, Assam and Cambod types of tea plants [Camellia sinensis (L.) O. Kuntze]
Source: Plant Methods. 2023 Dec 15;19:147. doi: 10.1186/s13007-023-01120-z (PMC10724972; doi:10.1186/s13007-023-01120-z)
Supplement: Supplementary file 1 — Supplementary Material 1: Important features to distinguish different tea cultivars used in the study [file 13007_2023_1120_MOESM1_ESM.docx]

**Table S1.** Important features to distinguish different tea cultivars used in the study

| **Accession No.** | **Accession Code** | **Type** | **Source** | **Location in Banuri tea farm** | ***Unique features** |
| --- | --- | --- | --- | --- | --- |
| IHBT-117 | CEF-02  (Him Spurthi) | Chinary; semi-arbour | Tea Experimental Farm, Banuri, CSIR-IHBT | Germplasm Block F | Flushing time: Early  Mature leaf type: Large, dark green, wavy and serrulate,  smooth leaf surface, acute recurved apex  Internode length: 2.27 cm  Two and a bud trait: Green colour, sparse pubescence  Shoots density: Dense  Floral traits: Cluster of flowers in axil, androecium higher than gynoecium |
| IHBT-122 | Kangra Asha | Chinary; shrub | CSK HPKV, Palampur | Germplasm Block A | Flushing time: Mid-season flush  Mature leaf type: Medium, dark green, ovate, biserrate,  smooth leaf surface, blunt tip, down turned  Internode length: 2.92 cm  Two and a bud trait: Purple colour, sparse pubescence  Shoots density: Dense  Floral traits: Cluster of flowers at cluster of flowers  in axilend of branches, gynoecium is higher than androecium |
| IHBT-176 | TV-23 | Cambod; semi-arbour | Tea Research Association (TRA), Tocklai, Assam | Germplasm Block A | Flushing time: Mid-season flush  Mature leaf type: Large, yellow-green, elliptic, serrulate  margin, leaf apex acute straight,  smooth leaf surface  Internode length: 1.99 cm  Two and a bud  traits: Green colour, intermediate pubescence  Shoots density: Dense  Floral traits: Cluster of flowers in axil, gynoecium  higher than androecium |
| IHBT-182 | UPASI-09 | Assamica; semi-arbour | Brookland Tea Estate, Valparai, Tamil Nadu | Germplasm Block A | Flushing time: Mid-season flush  Mature leaf type: Medium-large, green, biserrated  margin, leaf apex acute recurved,  smooth leaf surface  Internode length: 3.45 cm  Two and a bud  traits: Green colour, sparse pubescence  Shoots density: Intermediate  Floral traits: Cluster of flowers in axil, androecium is higher than Gynoecium |

*Source: Singh S, Kumar A. Tea germplasm at CSIR-IHBT. 2020 (https://library.ihbt.res.in/Institute%20Brochures/TEA%20GERMPLASM%20AT%20CSIR%20IHBT%20BOOK.pdf)
